# Lysosomal checkpoints in renal autoimmunity: from antigen processing to metabolic-immune crosstalk

**DOI:** 10.3389/fimmu.2026.1830023

**Published:** 2026-04-29

**Authors:** Zhengjing Fu, Xiang Xu

**Affiliations:** Changzhou De’an Hospital, Changzhou, Jiangsu, China

**Keywords:** antigen processing, immune tolerance, lupus nephritis, lysosomal checkpoint, metabolic reprogramming, renal autoimmunity

## Abstract

Lysosomes serve as critical intracellular hubs for degradation and signaling, playing a central role in maintaining immune homeostasis. In recent years, lysosomal functions have been conceptualized as a series of “checkpoints” that finely regulate the initiation and progression of autoimmune kidney diseases. This review systematically examines the multifaceted roles of lysosomal checkpoints in renal autoimmunity, with a focus on their mechanisms in autoantigen processing and presentation, immune cell activation and breakdown of tolerance, and the emerging area of metabolic-immune crosstalk. By integrating the latest research, this article aims to elucidate the potential of targeting lysosomal pathways as a novel therapeutic strategy for autoimmune kidney diseases such as lupus nephritis and ANCA-associated vasculitis.

## 1 Introduction

Kidney autoimmune diseases, such as lupus nephritis and ANCA-associated vasculitis, involve complex immune dysregulation in their pathogenesis. While traditional research has focused on the aberrant activation of the adaptive immune system, recent advances highlight the pivotal role of innate immune cells and renal parenchymal cells, where lysosomal function emerges as a critical regulator of disease progression. Lysosomes are no longer viewed merely as degradation organelles; their functional state—encompassing acidification, enzymatic activity, membrane permeability, and interactions with other organelles—constitutes a series of dynamic “checkpoints” that profoundly influence the fate of autoantigens, inflammasome activation, cellular metabolic status, and ultimately, the direction of immune responses. Understanding how these lysosomal checkpoints integrate environmental signals, metabolic cues, and immune instructions is crucial for unraveling the pathogenesis of kidney autoimmunity and developing precision therapies.

The integration of lysosomal function with innate immune signaling represents a fundamental checkpoint in kidney autoimmunity. Lysosomes serve as hubs for pathogen sensing and antigen processing, with their enzymatic activity directly modulating immune activation. For instance, cathepsins, key lysosomal proteases, are involved in processing antigens and regulating cytokine production. In teleost models, cathepsin L (CTSL) demonstrates proteolytic activity against bacteria and upregulates innate immune genes such as IL-1β, IL-6, and IFNγ, indicating its role in bridging lysosomal degradation with immune activation (1). Similarly, cathepsin C (CTSC) contributes to antibacterial immunity by promoting proinflammatory cytokine expression, including IL-1β and TNF-α, and its activity is influenced by lysosomal pH and ion concentrations (2). Beyond proteases, lysosomal transporters like SLC15A4 are essential for type-I interferon production in plasmacytoid dendritic cells and B cell functions downstream of TLR7/9 signals, with its deficiency protecting murine models from lupus-like disease (3). This underscores how lysosomal solute homeostasis, maintained by transporters such as SLC29A3, supports phagosomal pH regulation and anti-bacterial signaling in dendritic cells, linking lysosomal integrity to effective antigen presentation and autophagy induction (4). Moreover, the cGAS-STING pathway, a critical DNA-sensing mechanism, is regulated by lysosomal degradation; UNC93B1 attenuates STING signaling by targeting it for autophagy-lysosome degradation, preventing hyperactivation that could drive autoimmunity (5). These examples illustrate that lysosomes act as gatekeepers of innate immune responses, where dysregulation of their enzymatic or transport functions can skew immune signaling toward inflammation, contributing to autoimmune pathogenesis in the kidney. While this review integrates mechanistic insights from diverse cellular and disease models to construct a comprehensive framework, we place specific emphasis on the available evidence directly derived from classic renal autoimmune disorders, particularly lupus nephritis and ANCA-associated vasculitis, highlighting where mechanistic principles await full validation in these specific contexts.

Lysosomal checkpoints also exert profound influence over cellular metabolism and death pathways, creating a metabolic-immune crosstalk that fuels kidney autoimmunity. The metabolic state of immune and renal cells is intricately linked to lysosomal activity, particularly through processes like autophagy and mitophagy, which maintain cellular homeostasis. In systemic lupus erythematosus, autophagy plays a dual role: while it can promote inflammation by activating innate and adaptive immunity, defective autophagy may accelerate kidney injury by impairing podocyte integrity and promoting fibrosis (6). Autophagy dysregulation is implicated in lupus nephritis, where hydroxychloroquine (HCQ) exerts therapeutic effects by alkalinizing lysosomes and inhibiting autophagic activity, thereby reducing inflammation and T cell activation (7). Beyond autophagy, lysosomes are central to ferroptosis, a form of regulated cell death driven by lipid peroxidation. In acute kidney injury models, STING promotes ferroptosis through NCOA4-mediated ferritinophagy, where ferritin is degraded in lysosomes, leading to iron overload and tubular damage (8). This links lysosomal degradation directly to metabolic stress and cell death in renal tubules. Additionally, mitophagy, the selective autophagy of mitochondria, is manipulated by pathogens to evade immunity; for example, influenza virus nucleoprotein induces mitophagy to degrade MAVS and block antiviral signaling, highlighting how lysosomal pathways can be hijacked to dampen immune responses (9). In kidney cells, maintaining lysosomal function is vital for quality control, as failures in lysosomal activity in renal tubular epithelial cells contribute to senescence, apoptosis, and fibrosis (10). Furthermore, lysosomal storage disorders like Fabry disease, characterized by Gb3 accumulation in lysosomes, trigger immune-mediated inflammation and oxidative stress, exacerbating kidney injury and demonstrating how lysosomal defects can perpetuate metabolic and immune dysfunction (11). Thus, lysosomes serve as metabolic integrators, where their dysfunction disrupts energy balance, promotes cell death, and amplifies inflammatory signals, thereby driving autoimmune pathology in the kidney.

The role of lysosomes extends to shaping adaptive immune responses and autoantigen presentation, positioning them as critical checkpoints in the breakdown of self-tolerance in kidney autoimmunity. Lysosomes are essential for processing antigens into peptides for major histocompatibility complex (MHC) presentation, a process that, when dysregulated, can lead to autoimmunity. In Atlantic salmon, the intestine shows high activity in antigen presentation and innate antiviral immunity, with lysosomal proteases playing a key role in degrading luminal and intracellular proteins for immune surveillance (12). This underscores the conserved function of lysosomes in antigen processing across species. In autoimmune contexts, lysosomal enzymes like cathepsin C (CTSC) activate serine proteases in immune cells, including neutrophil elastase and proteinase 3, which are targets of anti-neutrophil cytoplasmic antibodies (ANCA) in ANCA-associated vasculitis, a kidney autoimmune disease (13). CTSC variants are associated with inflammatory disorders, highlighting how lysosomal protease activity can influence autoantigen generation and neutrophil activation, driving vasculitis. Moreover, lysosomal integrity affects dendritic cell function, where SLC29A3 supports antigen presentation on MHC-II molecules and promotes autophagy to control inflammation, linking lysosomal transport to adaptive immunity (4). Dysfunctional lysosomal degradation can also lead to accumulation of autoantigens. For example, in Fabry disease, Gb3 accumulation in lysosomes triggers inflammatory responses and oxidative stress, with overlapping features with systemic lupus erythematosus, suggesting shared lysosomal pathways in autoimmunity (14). Additionally, hydroxychloroquine, used in lupus nephritis, inhibits lysosomal function and antigen presentation, reducing autoantibody production and modulating T cell responses (7). Lysosomal stress responses, including membrane damage, can release pathogenic molecules into the cytoplasm, activating inflammasomes and promoting inflammation, as seen in various autoimmune and metabolic diseases (15). In kidney autoimmunity, this may enhance the presentation of self-antigens, breaking tolerance. Furthermore, lysosomal defects in diseases like lysinuric protein intolerance cause tubular damage and immune dysfunction, illustrating how lysosomal impairments in renal cells can initiate autoimmune-like pathology (16). Thus, lysosomes act as pivotal checkpoints in antigen processing and immune cell regulation, where their dysfunction can precipitate adaptive immune activation against self-tissues, culminating in kidney autoimmune diseases.

In this review, we define a ‘lysosomal checkpoint’ as a specific functional node—encompassing acidification, proteolytic activity, membrane permeability, or signaling platform capacity—where dysregulation shifts the outcome from physiological homeostasis toward autoimmunity. We organize these checkpoints into three interconnected axes: (1) The Proteolytic Checkpoint, governing autoantigen generation and MHC loading; (2) The Signaling Checkpoint, controlling mTORC1/AMPK balance and inflammatory cascades; and (3) The Structural Checkpoint, concerning membrane integrity and inter-organellar communication. This framework aims to provide a systematic view of how lysosomal dysfunction integrates diverse pathogenic signals in the kidney.

## 2 Lysosomes as the central hub for antigen processing and presentation

### 2.1 Lysosomal degradation of autoantigens and loading onto MHC class II molecules

The initiation of renal autoimmune responses critically depends on the lysosomal processing of self-antigens released from damaged tissue, such as nucleosomes and myeloperoxidase, and their subsequent presentation via major histocompatibility complex class II (MHC-II) molecules on antigen-presenting cells (APCs). Following endocytosis, these autoantigens are delivered to the lysosomal compartment, where their degradation into immunogenic peptides is governed by specific lysosomal checkpoints. The acidic pH and the activity of lysosomal proteases, notably cathepsins, are fundamental determinants of this process. For instance, cathepsin S (CatS) plays a pivotal role in the lysosomal/endosomal pathway by degrading the invariant chain (CD74), a necessary step for the efficient loading of antigenic peptides onto MHC-II molecules (17). Dysregulation of this proteolytic activity can lead to incomplete antigen degradation or the generation of novel cryptic self-epitopes, potentially breaking immune tolerance. This is exemplified in systemic lupus erythematosus (SLE), where inhibition of CatS with ASP1617 was shown to suppress MHC-II surface expression, T and B cell activation, and the production of pathogenic autoantibodies like anti-dsDNA IgG in a lupus-prone mouse model, highlighting CatS as a key checkpoint whose dysfunction can promote autoreactivity (17). Beyond protease activity, the integrity and function of lysosomal membrane proteins are equally crucial. The lysosomal-associated membrane protein 2 (LAMP2) is essential for autolysosome maturation and, by extension, for the autophagy- lysosomal degradation pathway that contributes to self-antigen processing for MHC- II presentation (18). In the thymic epithelium, genetic inactivation of *Lamp2* impairs autophagy and alters MHC-II processing, leading to a marked reduction in the diversity of the selected CD4+ T cell receptor repertoire (18). This underscores how lysosomal membrane stability and the autophagy-lysosomal axis interconnect to control the generation of self-peptide:MHC-II complexes. Furthermore, in the context of kidney-specific autoimmunity, such as primary membranous nephropathy (PMN), the autoantigen phospholipase A2 receptor (PLA2R) undergoes antibody-induced, clathrin-mediated endocytosis and is trafficked to lysosomes for degradation (19). This process enhances MHC-II-mediated presentation of PLA2R-derived peptides, amplifying the activation of antigen- specific CD4+ T cells and potentially establishing a self-perpetuating autoimmune loop (19). Thus, the lysosome serves as a central processing hub where checkpoints involving acidity, proteolytic enzymes like cathepsins, and membrane proteins like LAMP2 and HLA-DM (implicitly involved in peptide editing) collectively determine the immunogenic outcome of autoantigen degradation, with dysregulation at any point posing a risk for the activation of autoreactive T cells and the propagation of renal autoimmune disease. Importantly, while the fundamental principles of lysosomal antigen processing are conserved, the generation of specific nephritogenic epitopes (e.g., from α3(IV)NC1 in Goodpasture’s disease or PLA2R in primary membranous nephropathy) provides direct, disease-relevant evidence for this checkpoint in renal autoimmunity.

### 2.2 Lysosomal escape mechanisms in cross-presentation

In the inflammatory milieu of the kidney, certain autoantigens or immune complexes can bypass the conventional MHC-II pathway and instead be presented on MHC class I molecules to activate CD8+ T cells, a process known as cross- presentation. This pathway is critical for initiating cytotoxic responses against intracellular pathogens and, in autoimmunity, can contribute to tissue-specific damage. A key step in cross-presentation involves the escape of internalized antigens from the endosomal/lysosomal system into the cytosol, where they become substrates for proteasomal degradation and subsequent loading onto MHC-I molecules. The stability of the lysosomal membrane is therefore a vital checkpoint controlling this escape mechanism. Disruption of lysosomal integrity, whether through physiological processes or pathological insults, can facilitate antigen leakage into the cytosol and potentially exacerbate autoimmune injury. While the provided references do not directly detail cross-presentation of renal autoantigens, they offer foundational principles of lysosomal processing and antigen fate determination that are relevant to this concept. For example, the study on sulfatide analogs demonstrates how lysosomal processing can fundamentally alter the immunological fate of an antigen, switching its presentation from stimulating one lymphocyte subset (type II NKT cells) to another (type I NKT cells) (20). This “re- routing” was abrogated by inhibiting lysosomal acidification with bafilomycin A1 or by blocking the lysosomal enzyme arylsulfatase, proving that the lysosomal compartment actively reshapes antigen identity and determines the specificity of the ensuing immune response (20). This principle can be extrapolated to cross- presentation, where the extent and nature of lysosomal processing influence whether an antigen remains sequestered for MHC-II presentation or is channeled into the cytosolic pathway for MHC-I presentation. Furthermore, the interconnection between autophagy and antigen presentation pathways highlights alternative routes for cytosolic antigen access. Macroautophagy delivers cytosolic contents, including potential autoantigens, to lysosomes for degradation, and antigens processed via this pathway can be presented on both MHC-II and, under certain conditions, MHC-I molecules (21). The autophagy-lysosomal axis, regulated by proteins like LAMP2, thus represents a cellular mechanism that bridges endogenous antigen sources with the presentation machinery (18). In a renal autoimmune context, impaired autophagy or lysosomal membrane instability could lead to aberrant delivery of self-antigens to the cytosol, promoting their cross- presentation and activation of autoreactive CD8+ T cells. Although direct evidence for lysosomal escape of specific renal autoantigens like myeloperoxidase complexes is not provided here, the established role of lysosomal stability as a gatekeeper for antigen trafficking supports the model that its dysregulation serves as a checkpoint that can tilt the balance from tolerance to destructive CD8+ T cell-mediated autoimmunity in the kidney.

## 3 Lysosomal checkpoint regulates the activation and function of innate immune cells

### 3.1 Dendritic cell maturation and tolerance breakdown

Lysosomal activity directly regulates the maturation state of dendritic cells (DCs), a process central to the initiation of adaptive immunity and the maintenance of peripheral tolerance. High lysosomal activity is typically associated with a tolerogenic DC phenotype, which is crucial for preventing autoimmunity. Conversely, alterations in lysosomal function, often driven by signals such as those from Toll- like receptors (TLRs), can propel DCs towards a mature, immunogenic state capable of presenting self-antigens and breaking tolerance. This delicate balance is governed by intricate intracellular signaling networks. For instance, the molecule Optineurin (OPTN) is upregulated during human and mouse DC maturation and functions by binding to JAK2, inhibiting its dimerization and phosphorylation, thereby suppressing the JAK2-STAT3-IL-10 signaling axis (22). Without this negative regulation, as seen in *Optn*-deficient DCs, an IL-10/JAK2/STAT3 positive feedback loop is established, which paradoxically suppresses DC maturation and ameliorates autoimmune symptoms like experimental autoimmune encephalomyelitis (EAE) (22). Furthermore, the metabolic state of DCs is a key determinant of their function. The metabolite itaconate exerts anti-inflammatory effects in autoimmune hepatitis (AIH) by decreasing glycolysis, which in turn suppresses the maturation of DCs in the liver and spleen, thereby regulating the balance between pro-inflammatory Th17 cells and regulatory T cells (Tregs) (23). This suppression of maturation is linked to the inhibition of autophagy via activation of the PI3K/AKT/mTOR signaling pathway (23). The mTORC1 pathway itself is a core regulator of DC metabolism and function. Its overactivation can disrupt peripheral tolerance and promote the differentiation of pro-inflammatory DCs. Supporting this, in AIH, aberrant autophagy flux, a process closely linked to lysosomal function and regulated by pathways like mTOR, plays a vital role in the excessive, immunogenic maturation of conventional DCs (cDCs) (24). Pharmacological inhibition of this autophagy flux can induce a tolerogenic cDC phenotype, suggesting a therapeutic avenue (24). The breakdown of tolerance is also facilitated by external cellular interactions. Activated γδ T cells can promote DC maturation by secreting interferon-γ (IFN-γ), leading to increased expression of co-stimulatory molecules (CD80, CD83, CD86) and adhesion molecules (ICAM-1), which exacerbates autoimmune conditions like experimental autoimmune uveitis (EAU) by enhancing Th1/Th17 cell generation (25). Conversely, regulatory cell populations can enforce tolerance. Myelin-reactive CD8+ T cells can induce conventional DC subsets (cDC1 and CD11b+ cDC) to adopt a mature yet regulatory phenotype with an anti- inflammatory cytokine profile, which inhibits pathogenic CD4+ T cell responses and suppresses EAE (26). This highlights that maturation does not exclusively equate to immunogenicity; it can be coupled with regulatory functions. Therapeutic interventions often aim to modulate DC maturation. The natural product Total Glucosides of Paeony (TGP) ameliorates AIH by decreasing hepatocyte apoptosis and inhibiting DC maturation (27). Similarly, Astragaloside IV (ASI) alleviates EAE by suppressing the maturation and antigen-presentation capacity of DCs, evidenced by decreased expression of CD11c, CD86, CD40, and MHC II, and by inhibiting the downstream differentiation of Th1 and Th17 cells (28). The administration of soluble PD-L1 (sPD-L1) protein also ameliorates EAE by restraining DC maturation and migration, as shown by inhibited expression of CD86 and CCR7, and by mitigating DC morphological maturation and biomechanical alterations (29). Even cellular therapies, such as co-culture with human periodontal ligament stem cells (PDLSCs), can exert immunosuppressive effects on mature DCs from patients with type 1 diabetes, reducing the expression of key maturation markers (CD80, CD83, CD86, CD40, HLA-DR) (30). Nutritional factors like vitamins D and E can also regulate DC function and maturation, playing immunoregulatory roles (31). A novel mechanism of inducing antigen-specific tolerance involves the maturation of circulating Ly6C(^hi)CCR2(^+) monocytes into Ly6C(^hi)MHCII(^+)PD-L1(^+) cells by oxidized mannan-conjugated myelin peptides, which reverses established autoimmune encephalomyelitis (32). Furthermore, bile acids like lithocholic acid (LCA) can inhibit DC maturation and attenuate EAU severity by activating the TGR5 receptor, which downregulates NF-κB and MAPK pathways by reducing intracellular glutathione and inducing oxidative stress (33). The lysosome-associated mTORC1 signaling is further implicated, as demonstrated in AIH where itaconate’s action via the PI3K/AKT/mTOR pathway inhibits DC maturation and autophagy (23). In lupus- like disease, B cell-activating factor (BAFF) production from dendritic cells, monocytes, and neutrophils is required for B cell maturation and autoantibody production, linking myeloid cell function to humoral autoimmunity (34). These findings collectively underscore that lysosomal and associated metabolic checkpoints, particularly through mTORC1, are pivotal in determining whether DCs promote immune tolerance or instigate autoimmune pathology.

### 3.2 Macrophage polarization and inflammasome activation

Lysosomal function critically influences macrophage polarization towards classically activated pro-inflammatory (M1) or alternatively activated repair- associated (M2) phenotypes, thereby shaping the inflammatory landscape in tissues like the kidney. A key mechanism linking lysosomes to inflammation is the activation of the NLRP3 inflammasome. The leakage of lysosomal contents, such as cathepsins, into the cytosol can serve as a danger signal that activates the NLRP3 inflammasome, leading to the cleavage of pro-IL-1β and pro-IL-18 into their active forms and subsequent release of these potent inflammatory cytokines. This process is a significant driver of sterile inflammation in various contexts. The integrity of the lysosomal membrane acts as a crucial physical checkpoint to prevent this aberrant inflammasome activation. Breach of this integrity, often due to lysosomal membrane permeabilization (LMP), is a common trigger. While the provided references focus extensively on dendritic cell (DC) biology, the principles of lysosomal content leakage and inflammasome activation are well-established in macrophage immunology. For instance, in the context of renal fibrosis, a process driven by chronic inflammation, dendritic cells exacerbate pathology through metabolic reprogramming and crosstalk with renal tubular epithelial cells (RTECs) (35). Although this study highlights DCs, macrophages are equally central to renal inflammation and fibrosis, and similar lysosomal/inflammasome pathways are operational. Hydroxychloroquine (HCQ), known to accumulate in lysosomes and alter their pH, alleviates renal fibrosis by modulating DC glycolipid metabolism and impairing DC-RTEC crosstalk (35). Its mechanism likely involves stabilizing lysosomal membranes, thereby preventing content leakage and subsequent inflammatory signaling, an action that would also be relevant to macrophages in the kidney. Furthermore, the crosstalk between metabolic states and immune activation is pivotal. In cancer therapy, strategies that induce immunogenic cell death (ICD) often involve processes that trigger lysosomal damage or oxidative stress, leading to the release of damage-associated molecular patterns (DAMPs) which promote DC maturation (36). This paradigm is directly applicable to macrophages; for example, inducing ferroptosis in tumor cells releases DAMPs that can reprogram tumor-associated macrophages (TAMs) and influence their polarization (36). The metabolite 5’-deoxy-5’methylthioadenosine (MTA), which accumulates in the tumor microenvironment, impairs the maturation of monocyte-derived DCs, reducing their T cell-stimulating capacity (37). This suggests that tumor metabolites can modulate myeloid cell function, potentially including macrophage polarization, through mechanisms that may involve lysosomal or metabolic dysregulation. Edible fungal polysaccharides, recognized immunomodulators, engage core signaling pathways such as the NLRP3 inflammasome to regulate macrophage polarization and DC maturation (38). This directly connects lysosome-sensitive inflammasome activation to the functional fate of myeloid cells. In hepatocellular carcinoma (HCC), the HDAC inhibitor romidepsin confers an immune-stimulatory profile, with direct effects on primary human dendritic cell maturation *in vitro* (39). While focused on DCs, HDAC inhibitors are also known to affect macrophage polarization, often through epigenetic regulation of inflammatory genes, some of which are downstream of inflammasome activation. The cGAS-STING pathway, activated by cytosolic DNA (which can be released from damaged organelles including mitochondria, a process sometimes linked to lysosomal dysfunction), is another key inflammatory cascade. In a nanoplatform designed for bladder cancer, Zn2+ release triggers mitochondrial DNA leakage, activating the cGAS-STING pathway to drive DC maturation (40). This pathway is also critically involved in macrophage activation and polarization. Therefore, maintaining lysosomal membrane integrity is essential to compartmentalize hydrolytic enzymes and prevent the uncontrolled release of contents that can activate the NLRP3 inflammasome or other cytosolic sensors like cGAS. Failure of this checkpoint, due to factors like crystalline substances, oxidative stress, or certain lipids, may lead to IL-1β-driven inflammation, a hallmark of many sterile inflammatory diseases including crystal-induced nephropathy, and could potentially contribute to the inflammatory milieu in autoimmune kidney diseases. The interplay between lysosomal stability, metabolic cues (like cholesterol dynamics or glycolipid metabolism), and inflammasome signaling forms a critical axis determining macrophage effector functions and the progression of tissue inflammation and fibrosis.

## 4 Lysosomes and immunogenic death of renal parenchymal cells and release of damage signals

The following section examines lysosomal-dependent injury and signaling in renal parenchymal cells. While the specific triggers in models such as toxic nephropathy or metabolic stress differ from the primary loss of tolerance seen in lupus nephritis, these models illuminate a critical secondary principle: lysosomal dysfunction within podocytes and tubular cells acts as an endogenous adjuvant, releasing damage signals and autoantigens that can initiate or markedly exacerbate immunogenic inflammation. These mechanisms are highly relevant to understanding how established autoimmunity translates into organ-specific damage in the kidney.

### 4.1 Podocyte and renal tubular epithelial cell lysosome-dependent injury

Under autoimmune attack, the lysosomes in glomerular podocytes and renal tubular epithelial cells undergo leakage, releasing cathepsins and other contents into the cytoplasm, which induces pyroptosis or apoptosis, further exposing intracellular autoantigens. Lysosomal membrane protein (e.g., LAMP1/2) expression changes can serve as markers for cellular damage and enhanced immunogenicity. In glomerular diseases such as focal segmental glomerulosclerosis (FSGS), podocyte-specific injury models demonstrate that lysosomal stress response is among the earliest detectable alterations, preceding significant structural damage (41). This lysosomal stress, characterized by increased lysosomal abundance and galectin-3 expression, is closely associated with mitochondrial injury and the initiation of inflammatory signaling, including elevated chemokine CXCL1 expression within the podocyte (41). Lysosomes in podocytes are not merely degradative organelles but also serve as platforms for cellular signaling, and their dysfunction is a critical pathogenic factor in chronic glomerular diseases (42). For instance, in obesity-related glomerulopathy, impaired lysosomal function, such as reduced lysosome-multivesicular body (MVB) interaction, contributes to NLRP3 inflammasome activation and subsequent podocyte injury (43). Lysosomal leakage or dysfunction can trigger various forms of programmed cell death. In Fabry disease, a lysosomal storage disorder, the accumulated metabolite lyso-Gb3 induces podocyte death, partly through pathways involving RIPK3, which is associated with reactive oxygen species generation and cytoskeleton rearrangement (44). Similarly, exposure to toxic substances like chloroquine, which mimics lysosomal storage conditions, induces lysosomal accumulation and morphological alterations in podocytes, including changes in F- actin and nucleus structure, linking lysosomal dysfunction to integrin modulation and cell injury (45). The release of lysosomal enzymes into the cytosol can also disrupt the delicate balance between the ubiquitin-proteasome system (UPS) and the autophagy-lysosomal system (ALS). Impairment of proteasome function in podocytes leads to the accumulation of ubiquitinated and oxidatively modified proteins, which can induce apoptosis; notably, this impairment may also suppress compensatory autophagic activity, exacerbating cell injury (46). In renal tubular epithelial cells (TECs), lysosomal dysfunction is a central mechanism of injury across various etiologies. In hyperuricemia-induced injury, high uric acid causes lysosome dysfunction, impairing autophagy and leading to mitochondrial abnormalities, NLRP3-IL-1β inflammation, and apoptosis in proximal tubular epithelial cells (PTECs) (47). This is further evidenced by findings in patient exosomes showing activation of lysosomal processes and association of lysosomal- associated membrane protein-2 (LAMP2) expression with renal dysfunction (47). In drug-induced nephrotoxicity, such as with cisplatin, lysosomal damage and impaired autophagic flux are key contributors to tubular cell apoptosis and senescence (48). Cisplatin injury is exacerbated by interleukin-11 (IL-11), which disrupts lysosomal maturation and autophagic flux by suppressing transcription factor EB (TFEB) activity, linking lysosomal dysfunction to mitochondrial fission and cell death (48). Furthermore, chaperone-mediated autophagy (CMA), a selective lysosomal degradation pathway, can be hijacked in injury; for example, in antimony- induced nephrotoxicity, CMA activation leads to the lysosomal degradation of glutathione peroxidase 4 (GPX4), precipitating ferroptosis in renal tubular epithelial cells (49). Changes in lysosomal membrane proteins like LAMP1 and LAMP2 are indicative of this damage. In models of contrast-induced acute kidney injury (CI- AKI), inhibition of the NLRP3 inflammasome upregulates mitophagy and is associated with increased LAMP1, suggesting a role in organelle clearance (50). In acute oxalate nephropathy, inhibition of HDAC6 reduces inflammation and necroptosis, processes intertwined with lysosomal exocytosis pathways (51). Therefore, lysosomal leakage and the resultant shift in the degradative equilibrium not only directly compromise cell viability but may also amplify immunogenicity by exposing intracellular contents, with LAMP1/2 serving as potential sentinels of this injury cascade.

### 4.2 Extracellular vesicles as carriers of lysosome-derived immune signals

Dysfunctional renal cells can release extracellular vesicles (EVs) containing lysosomal components (e.g., enzymes, RNA). These vesicles, upon uptake by neighboring immune cells, can transmit inflammatory or autoantigen signals, amplifying the local immune response. In podocytes, activation of the NLRP3 inflammasome leads to the formation of IL-1β-containing multivesicular bodies (MVBs), and the subsequent release of EVs is a key mechanism for propagating inflammatory signals (43). Adiponectin receptor agonists protect against glomerular inflammation in obesity-related kidney disease by suppressing this visfatin-induced EV release from podocytes, an effect dependent on restoring lysosome-MVB interactions via acid ceramidase activation and TRPML1 channel-mediated Ca2+ release (43). This highlights a direct link between lysosomal function, EV biogenesis, and immune signal dissemination. EVs serve as vehicles for lysosome-derived cargo that can modulate recipient cell behavior. In hyperuricemia, urinary exosomes from patients show activated lysosomal processes, and exosomal expression of lysosomal-associated membrane protein-2 (LAMP2) correlates with disease severity, suggesting that EVs may carry markers of lysosomal stress from tubular cells into the urine, potentially serving as both biomarkers and intercellular messengers (47). The cargo within these EVs can include not only proteins but also nucleic acids and lipids that reflect the lysosomal and autophagic status of the parent cell. In diabetic kidney disease (DKD), impaired autophagy and lysosomal function in renal cells are hallmarks, and restoring autophagic flux—a process culminating in lysosomal degradation—is a therapeutic target for various agents, including Chinese herbal compounds (52). It is plausible that EVs released from autophagy-deficient podocytes or tubular epithelial cells carry undegraded substrates, DAMPs (damage- associated molecular patterns), or specific miRNAs that can activate immune cells upon uptake. For instance, in acute kidney injury (AKI) models, macrophage autophagy plays a protective role by degrading pro-inflammatory mediators like TARM1 via the autophagy-lysosome pathway, thereby curbing renal inflammation (53). Conversely, autophagy deficiency in macrophages could alter the EV profile they release, potentially exacerbating inflammation. Furthermore, in chronic settings like renal fibrosis, macrophage-specific deletion of Atg5 impairs migration via the CCL20-CCR6 axis but also reduces fibrosis, indicating that autophagy in immune cells regulates their recruitment and signaling, processes likely influenced by EV-mediated communication (54). EVs derived from injured tubular cells under oxidative stress, such as in cisplatin-induced AKI, may contain mitochondrial components or oxidized proteins due to defective lysosomal clearance, which could act as immunogenic signals when internalized by macrophages or dendritic cells (55). Similarly, in lipotoxicity models of obesity-related kidney disease, lysosomal dysfunction and phospholipid accumulation in proximal tubular epithelial cells are features of lipotoxicity, and enhancing lysosomal exocytosis via TFEB can alleviate this burden (56). This lysosomal exocytosis is a mechanism for expelling excess lipids and potentially other materials into EVs, which could then interact with immune cells in the interstitium. The role of EVs as carriers is also implicated in the progression of diabetic kidney disease, where lysosomal dyshomeostasis in podocytes and tubular cells contributes to injury, and strategies to maintain lysosomal function may reduce the release of pathogenic EVs (57). In Fabry disease, nanoparticles that enhance autophagy flux reduce globotriaosylceramide (Gb3) accumulation and attenuate kidney injury, suggesting that improving lysosomal function may also normalize EV secretion profiles, reducing the delivery of storage material or inflammatory signals (58). Therefore, extracellular vesicles act as critical vectors in the metabolic-immune crosstalk within the kidney, transporting lysosome-derived components from injured resident cells to immune cells, thereby perpetuating and amplifying the inflammatory response that characterizes autoimmune and metabolic kidney diseases.

## 5 Role of lysosome-mediated metabolic-immune crosstalk in renal autoimmunity

### 5.1 Lysosome as a metabolic sensing hub: the balance of mTORC1 and AMPK

The lysosomal membrane serves as a critical platform for the mammalian target of rapamycin complex 1 (mTORC1) signaling pathway, enabling cells to sense nutrient status, particularly amino acid availability (59). This positioning allows mTORC1 to integrate signals from growth factors and energy levels to regulate anabolic processes, cell survival, and proliferation. In the context of active renal autoimmunity, such as in systemic lupus erythematosus (SLE) and lupus nephritis, dysregulation of this sensing mechanism is frequently observed. For instance, in SLE T cells, the accumulation of cytosolic mitochondrial DNA (mtDNA) triggers a metabolic shift towards enhanced glycolysis via ENPP1. This shift depletes free fatty acids, impairing the N-myristoylation and subsequent lysosomal localization of AMP-activated protein kinase (AMPK) (60). The resultant inactivation of AMPK fails to restrain mTORC1, leading to its hyperactivation. This mTORC1 overdrive promotes a mal-differentiation of effector T cells towards a pro-inflammatory phenotype, contributing to autoantibody production and disease progression in lupus nephritis models (60). This illustrates how the lysosomal platform, intended for metabolic homeostasis, becomes a site of pathogenic signaling imbalance in autoimmunity.

Concurrently, AMPK acts as a central energy sensor that antagonizes mTORC1 activity and promotes catabolic processes, including lysosomal biogenesis and autophagy. AMPK activation is intricately linked to lysosomal function. Under low glucose conditions, the AXIN: LKB1 complex translocates to the lysosomal surface, where it facilitates AMPK activation and concomitant mTORC1 inhibition (61). This cross-talk is essential for adapting cellular metabolism to energy stress. However, in metabolic and immune disorders, AMPK activity is often compromised. In lipotoxic conditions relevant to metabolic renal disease, exposure of proximal tubular epithelial cells (PTECs) to palmitate leads to lysosomal deacidification and dysfunction. Preventing the palmitate-induced decline in AMPK activity with pharmacological activators preserves lysosomal acidification and PTEC differentiation, highlighting AMPK’s protective role against lysosomal stress (62). Furthermore, specific regulatory mechanisms exist; for example, mTORC1-mediated phosphorylation of AMPKα2 at Ser345 controls its lysosomal targeting for activation by LKB1, revealing an α2-specific phospho-switch that links mTORC1 feedback to AMPK activation at the lysosome independent of cellular energy changes (63). Impairment of this delicate AMPK/mTORC1 balance, as seen in autoimmune T cells or metabolically stressed renal cells, disrupts autophagic flux and quality control, creating a permissive environment for inflammation and tissue injury. Therefore, the lysosome stands as a pivotal metabolic sensing hub where the equilibrium between anabolic (mTORC1) and catabolic (AMPK) signals dictates cellular fate, and its dysregulation is likely a fundamental node in the immunometabolic dysfunction observed in kidney autoimmunity.

### 5.2 Immunomodulatory functions of the autophagy-lysosome pathway

Autophagy, a conserved lysosomal degradation pathway, functions as a crucial cellular quality control mechanism by clearing damaged organelles, misfolded proteins, and intracellular pathogens. This process directly modulates immune responses by degrading pro-inflammatory signaling molecules, such as components of the inflammasome, thereby suppressing excessive immune activation (64). The initiation and regulation of autophagy are coordinately controlled by cellular nutrient sensors, primarily AMPK and mTORC1. AMPK promotes autophagy initiation by phosphorylating ULK1 and inhibiting mTORC1, while mTORC1 suppresses autophagy under nutrient-rich conditions (59). Furthermore, the transcriptional activation of key regulators like TFEB and TFE3, which drive the expression of autophagy and lysosomal genes, requires AMPK-dependent phosphorylation, adding another layer of regulation to the autophagic response (65). Thus, a functional autophagy-lysosome pathway is essential for maintaining cellular homeostasis and preventing the accumulation of inflammatory substrates.

In lupus nephritis, a hallmark of systemic autoimmune kidney disease, pervasive defects in autophagy are observed in both immune cells and renal parenchymal cells. In T cells from SLE patients, the metabolic dysregulation driven by cytosolic mtDNA and ENPP1 leads to impaired AMPK activity and consequent mTORC1 hyperactivation, which suppresses autophagy (60). This autophagy deficiency contributes to the accumulation of damaged cellular components and inflammatory mediators. Similarly, in renal cells, lipotoxicity from fatty acids like palmitate can induce lysosomal dysfunction, characterized by impaired acidification, which blocks autophagic flux and activates lysosomal biogenesis pathways as a compensatory mechanism (62). When this compensation fails, it drives cellular dedifferentiation and injury. The accumulation of autophagy substrate p62/SQSTM1, resulting from impaired degradation, can itself exacerbate inflammation. p62 accumulation potentiates mTORC1 signaling and can also activate antioxidant responses via NFE2L2/NRF2, creating complex feedback loops during metabolic stress (66). In the context of autoimmunity, defective autophagy in T cells and renal cells leads to the inadequate clearance of endogenous nucleic acids and other potential autoantigens, as well as the hyperactivation of inflammasomes. This creates a vicious cycle: autoantigen accumulation and persistent inflammation further stress the autophagy-lysosome system, while its impairment amplifies immunogenic signals. Therefore, the immunomodulatory function of autophagy is critically compromised in lupus nephritis, where its failure to curb inflammatory processes and maintain cellular hygiene is thought to directly fuel disease pathogenesis by allowing the persistence of inflammatory stimuli and promoting a maladaptive immune response.

## 6 Hereditary and acquired lysosomal dysfunction and susceptibility to renal autoimmunity

### 6.1 Autoimmune phenomena associated with lysosomal storage disorders

Certain lysosomal storage disorders (LSDs), such as Gaucher disease and Fabry disease, are frequently accompanied by kidney damage that resembles autoimmune pathologies, suggesting that underlying lysosomal functional defects can directly or indirectly disrupt immune tolerance. The mechanisms involve the accumulation of undegraded substrates triggering chronic inflammation, altering antigen presentation, or directly injuring renal cells. For instance, in Gaucher disease, deficient glucocerebrosidase activity leads to glycosphingolipid accumulation within the mononuclear phagocyte system, which is associated with a proinflammatory state, cytokine and chemokine release, and potential autoimmune antibody production that can affect endocrine profiles and organ function, including the kidneys (67, 68). This chronic inflammatory milieu, driven by substrate storage, may compromise cellular homeostasis and disturb autophagy, processes central to maintaining immune balance (69, 70). Similarly, Fabry disease, caused by alpha- galactosidase deficiency and globotriaosylceramide (Gb-3) accumulation, is characterized by a potentiated production of pro-inflammatory mediators, revealing an acute inflammatory process that can become chronic and impact renal cells (71). The lysosomal dysfunction inherent to LSDs disrupts critical cellular processes such as metabolism, autophagy, and immune response regulation, which are pivotal for glomerular health (72). This dysfunction can lead to altered antigen processing and presentation, as lysosomes are essential for these functions in immune cells (69). Furthermore, the accumulation of substrates like glycosaminoglycans in mucopolysaccharidoses or nucleic acids in other contexts can elicit innate immune responses through pathways like Toll-like receptor activation, contributing to inflammation and tissue damage, including in the kidneys (73, 74). Evidence also indicates that specific LSDs, such as alpha-mannosidosis and lysinuric protein intolerance, are included in the differential diagnosis of autoimmune disorders, and patients may present with autoimmune conditions, further supporting the link between lysosomal defects and immune dysregulation (75, 76). The resulting chronic inflammation and potential for autoantibody generation can directly damage renal cells, as seen in cases where Fabry disease overlaps with systemic lupus erythematosus, leading to complex nephropathy (14). Thus, the fundamental lysosomal impairment in LSDs creates a permissive environment for breaking immune tolerance through intertwined metabolic, inflammatory, and antigen- presenting pathways, culminating in autoimmune-like kidney injury.

### 6.2 Perturbation of lysosomal checkpoints by environmental factors

Environmental factors such as infections, drugs (e.g., certain chemotherapeutic agents), or uremic toxins can impair lysosomal function, for example, by inhibiting V-ATPase to affect acidification or increasing membrane instability, thereby potentially inducing or exacerbating kidney autoimmune responses. Lysosomes are central signaling nodes that sense and orchestrate cellular metabolism and immune responses, and their function is tightly regulated by ion channels, transporters, and membrane integrity (72, 77). Environmental insults can disrupt this delicate homeostasis. For instance, cationic amphiphilic drugs, a class that includes some chemotherapeutic agents, can accumulate in lysosomes, perturb membrane permeability, and inhibit lysosomal hydrolases, leading to a form of drug-induced lysosomal storage disorder and dysfunction (77). Such drug-induced lysosomal damage can result in the spillage of proteolytic enzymes into the cytoplasm, causing cellular damage and activating inflammatory pathways, including the NLRP3 inflammasome, which is implicated in autoimmune pathologies (77, 78). Infections represent another major environmental perturbation. Pathogens or their components can be processed in lysosomes, and excessive influx or defective degradation of microbial nucleic acids can lead to lysosomal nucleic acid stress (74). This stress activates endosomal Toll-like receptors (e.g., TLR8) or cytoplasmic sensors (e.g., cGAS-STING, AIM2), initiating innate immune responses that, while protective, may also contribute to autoimmune and autoinflammatory diseases if dysregulated (74). Furthermore, in the context of kidney disease, uremic toxins accumulated in renal failure can directly impact lysosomal function. Although not explicitly detailed in the provided abstracts, the general principle that environmental toxins can alter lysosomal activity is supported by the role of lysosomes in responding to environmental changes and nutrient stresses (69). Such toxins could impair V-ATPase function, critical for maintaining the acidic pH essential for lysosomal enzyme activity, or increase oxidative stress, leading to lysosomal membrane damage (78). This lysosomal dysfunction, in turn, can deregulate autophagy—a process dependent on lysosomal ion channels and fusion events (77). Dysregulated autophagy is a hallmark of autoimmune disease and can contribute to inflammation by failing to clear damaged organelles or protein aggregates, leading to the release of damage-associated molecular patterns (DAMPs) (69, 77). In immune cells, impaired lysosomal function can alter antigen presentation and cytokine secretion, skewing immune responses (69). For example, in macrophages, lysosomal dysfunction can change cytokine secretion profiles, partially through inflammasome activation, promoting a pro-inflammatory state conducive to autoimmunity (79). Therefore, environmental factors may act as triggers that compromise lysosomal checkpoints, potentially fostering an inflammatory milieu and could unmask or aggravate autoimmune reactivity in the kidney.

## 7 Therapeutic strategies and prospects targeting lysosomal checkpoints

Therapeutic targeting of lysosomal checkpoints spans a spectrum of translational maturity, ranging from established clinical practice to emerging preclinical and future investigational strategies.

### 7.1 Pharmacological modulators: from hydroxychloroquine to novel inhibitors

#### 7.1.1 Clinically established

Hydroxychloroquine (HCQ) stands as a cornerstone pharmacological agent targeting the lysosomal checkpoint in renal autoimmunity, particularly in lupus nephritis (LN). As a classic lysosomotropic weak base, HCQ accumulates within acidic organelles like endosomes and lysosomes, where it neutralizes the luminal pH. This elevation of endosomal/lysosomal pH is its primary mechanism of action. By disrupting the acidic environment essential for optimal enzymatic activity, HCQ potently inhibits the proteolytic processing of autoantigens by lysosomal cathepsins. This interference with antigen processing and subsequent loading onto MHC class II molecules directly impairs the activation of autoreactive CD4+ T cells, a critical step in the pathogenesis of systemic lupus erythematosus (SLE) and LN. Furthermore, HCQ disrupts Toll-like receptor (TLR) signaling, especially endosomal TLRs like TLR7 and TLR9 which are crucial for sensing nucleic acids and driving interferon- alpha production in plasmacytoid dendritic cells. By alkalinizing the endosomal compartment, HCQ prevents the conformational changes and uncoupling of TLRs from their ligands, thereby blunting the innate immune activation that fuels autoantibody production. Its clinical efficacy and established safety profile make HCQ a successful paradigm for lysosomal checkpoint modulation, demonstrating that targeting organelle pH can have profound systemic immunomodulatory effects.

#### 7.1.2 Preclinical/investigational

Building on this foundational concept, research is actively pursuing novel, more specific agents. These include direct cathepsin inhibitors designed to block specific enzymes like cathepsin S or L involved in antigen presentation without broad pH disruption, potentially offering a better side-effect profile. Similarly, more selective inhibitors of the vacuolar-type H+-ATPase (V-ATPase), the proton pump responsible for lysosomal acidification, are under investigation to achieve targeted pH modulation. Another promising avenue involves lysosomal membrane stabilizers, which aim to prevent the pathological lysosomal membrane permeabilization (LMP) that can lead to the release of cathepsins and other hydrolases into the cytosol, triggering inflammation and cell death. These next-generation pharmacological modulators, currently in preclinical development, hold the potential for greater precision in correcting the specific lysosomal dysfunctions observed in different autoimmune kidney diseases, moving beyond the broad-spectrum approach of HCQ towards tailored therapeutic strategies.

### 7.2 Modulating autophagy and metabolic pathways

#### 7.2.1 Emerging clinical/preclinical strategies

Targeting the interconnected pathways of autophagy and cellular metabolism represents a highly promising strategy for treating renal autoimmune diseases by restoring lysosome-mediated homeostasis. Autophagy is a critical lysosomal degradation pathway for recycling damaged organelles, protein aggregates, and intracellular pathogens, and its dysregulation is implicated in the loss of immune tolerance. The mechanistic target of rapamycin (mTOR) is a central negative regulator of autophagy initiation. Pharmacological inhibition of mTOR with drugs like rapamycin (sirolimus) or its analogs can restore autophagic flux, promoting the clearance of endogenous autoantigens and mitigating endoplasmic reticulum stress in renal cells. This action helps re-establish cellular homeostasis and can suppress the aberrant activation of antigen-presenting cells and T cells. Concurrently, metabolic reprogramming is a hallmark of activated immune cells. In autoimmune settings, pathogenic lymphocytes often exhibit a shift towards aerobic glycolysis, a metabolic state that supports their proliferative and pro-inflammatory functions.

Correcting this metabolic imbalance is another therapeutic avenue. AMP-activated protein kinase (AMPK) is a key cellular energy sensor that promotes catabolic processes like autophagy and inhibits anabolic pathways. AMPK agonists, such as the widely used anti-diabetic drug metformin, can activate autophagy and shift cellular metabolism towards oxidative phosphorylation. By doing so, these agents can dampen the hyperactive metabolic state of autoreactive immune cells, reduce their survival and effector functions, and promote the generation of regulatory T cells. The therapeutic goal of using mTOR inhibitors or AMPK activators is not merely to induce autophagy but to correct a fundamental metabolic-immune imbalance. By restoring proper autophagic flux and normalizing cellular metabolism, these interventions aim to rebuild the lysosome’s role as a central hub for intracellular quality control and metabolic sensing. This re-established homeostasis can then suppress the cycle of abnormal immune activation, antigen presentation, and inflammation that drives tissue damage in conditions like lupus nephritis and other autoimmune kidney diseases, offering a path to disease modification rather than mere symptom suppression.

### 7.3 Prospects for gene and cell therapy

#### 7.3.1 Future prospects (investigational)

Looking towards the future of precision medicine for renal autoimmunity, advanced therapeutic modalities like gene and cell therapy offer promising directions by directly addressing the root causes of lysosomal dysfunction. For monogenic forms of autoimmune or autoinflammatory kidney disease where specific lysosomal gene defects are identified, gene correction therapies hold transformative potential.

Techniques such as CRISPR-Cas9 gene editing could be employed to correct mutations in genes encoding lysosomal hydrolases, membrane proteins, or transporters in patient-derived hematopoietic stem cells or specific renal cell types. By restoring normal lysosomal protein function, such therapies could prevent the accumulation of undegraded substrates, aberrant antigen processing, and inflammatory signaling that originate from the genetic lesion. This approach moves beyond managing symptoms to potentially offering a curative strategy for defined genetic subsets of disease. Complementing gene therapy, cell-based strategies focus on modulating the immune system by introducing cells with normalized lysosomal function. A prime example is the adoptive transfer of tolerogenic dendritic cells (tolDCs). These are dendritic cells engineered or conditioned *ex vivo* to have a stable regulatory phenotype, which is intimately linked to their lysosomal and metabolic state. TolDCs often exhibit altered lysosomal protease activity and antigen- processing capacity, favoring the presentation of tolerogenic peptides. Furthermore, their metabolic profile is shifted towards oxidative phosphorylation, supporting their long-term survival and regulatory function. Upon infusion, these tolDCs can migrate to lymphoid organs and tissues, where they can induce antigen-specific T cell anergy, deletion, or the generation of regulatory T cells, thereby re-establishing immune tolerance. The development of such therapies requires precise targeting of the lysosomal-autophagy-metabolism axis within specific immune cell subsets to ensure safety and efficacy. While significant challenges in delivery, specificity, and long-term stability remain, the convergence of insights into lysosomal biology with advances in genetic engineering and cell manufacturing paves the way for these next-generation treatments. They represent a paradigm shift from broad immunosuppression to targeted restoration of the lysosomal checkpoint’s physiological role in maintaining immune homeostasis within the kidney.

## 8 Challenges and future research directions

### 8.1 Cell type and disease stage specificity

The function of lysosomal checkpoints exhibits profound cell type and disease stage specificity, necessitating future research to utilize single-cell technologies to precisely delineate the dynamic alterations in lysosomal function across different renal cell populations (immune cells versus parenchymal cells) and distinct phases of disease (initiation, active, chronic). In immune cells, lysosomal activity is intricately linked to their functional state. For instance, in rheumatoid arthritis (RA), the generation of short-lived, tissue-invasive effector T cells is associated with underlying mitochondrial and lysosomal anomalies, highlighting a cell-intrinsic lysosomal checkpoint defect within a specific immune subset during disease pathogenesis (80). This dysfunction is part of a broader metabolic reprogramming that diverts glucose from energy production to support cell motility and building programs (80). Conversely, in parenchymal cells like osteoclasts, lysosomal function is regulated by distinct molecular checkpoints. Gasdermin D (GSDMD) acts as a critical brake on osteoclastic bone resorption by generating a non-lytic p20 fragment that localizes to early endosomes, limiting endo-lysosomal trafficking and maturation, thereby controlling excessive lysosomal activity and secretion (81). This checkpoint mechanism is essential for maintaining bone homeostasis, and its loss leads to enhanced bone resorption (81). The disease stage further modulates these checkpoints. Transcriptomic analyzes of viral infection models reveal that immune responses, including lysosome-related pathways, are dynamically regulated over time. In resistant organisms, efficient control of infection is associated with more active lysosome and proteasome functions at later stages, suggesting an adaptive enhancement of lysosomal degradation capacity as part of a sustained defense (82). In contrast, susceptible individuals may exhibit dysregulated early immune responses that fail to properly engage these degradative pathways (82). In cancer, the role of lysosomal components like Cathepsin C (CTSC) varies across tumor types and stages, with high expression correlating with poor prognosis and advanced stages in several cancers, indicating its stage-specific pro-tumorigenic functions (83). Similarly, in oral squamous cell carcinoma (OSCC), the lysosomal protease inhibitor cystatin-B (CSTB) is downregulated, and its loss is associated with poorer clinicopathological features like advanced tumor stage and lymph node metastasis, marking it as a factor whose dysregulation is significant in disease progression (84). In hepatocellular carcinoma (HCC), the lysosomal V-ATPase regulator ATP6AP1 drives an immunosuppressive tumor microenvironment, and its expression is associated with tumor stage, illustrating how a lysosome-related protein’s impact is contingent upon the disease’s advanced state (85). In kidney disease, the role of autophagy, a lysosome-dependent process, is highly context-dependent, varying by cell type and pathological circumstance, and can be either profibrotic or antifibrotic (86). For example, induction of autophagy in proximal tubular epithelial cells is protective against stress, whereas its loss increases susceptibility to injury (86). Therefore, a precise, single-cell resolution mapping of lysosomal gene expression, protease activity, and organelle integrity in renal resident macrophages, dendritic cells, T cells, podocytes, mesangial cells, and tubular epithelial cells across the temporal spectrum of autoimmune nephritis is imperative. This will identify which specific lysosomal checkpoints are compromised in which cells during initial loss of tolerance, active inflammatory damage, and the transition to chronic fibrosis, enabling the development of highly targeted therapeutic interventions.

### 8.2 Lysosomal interorganellar communication networks

A comprehensive understanding of lysosomal checkpoints in immunometabolic regulation requires in-depth investigation into the contact sites and communication between lysosomes and other organelles, including mitochondria (metabolism), the endoplasmic reticulum (stress), and the nucleus (epigenetics). Lysosomes are not isolated degradation centers but integral hubs within a dynamic interorganellar network. Their interaction with mitochondria is a key nexus for metabolic sensing and immune cell fate. In rheumatoid arthritis, defects in T cells involve concurrent mitochondrial and lysosomal anomalies, suggesting a coupled dysfunction where metabolic derangements (potentially from mitochondrial instability) culminate in lysosomal defects that drive aberrant effector cell generation (80). This lysosome-mitochondria axis is crucial for providing the metabolic platform for immune cell differentiation and function. Communication with the endoplasmic reticulum (ER) is vital for managing cellular stress. The process of autophagy, which delivers cytoplasmic cargo to lysosomes, is activated by various stresses, including those originating from the ER, and is essential for renal cell homeostasis (86). Furthermore, the transcription factor TGF-β1, which is activated in chronic kidney disease and can induce both fibrosis and autophagy, represents a signaling node that integrates extracellular cues with lysosomal biogenesis and function, indirectly linking lysosomal activity to broader transcriptional programs (86). A direct link to nuclear function and epigenetics is emerging through the regulation of lysosomal components themselves. The pan-cancer analysis of CTSC revealed that its expression correlates with DNA hypermethylation and RNA methylation regulators, indicating that lysosomal protease levels are subject to epigenetic control, which in turn influences the tumor immune microenvironment (83). Moreover, the GSDMD p20 fragment exerts its checkpoint function on osteoclast lysosomes by controlling phosphoinositide conversion, specifically by binding to phosphatidylinositol 3- phosphate (PI(3)P) on endosomal membranes (81). This highlights how lysosomal membrane dynamics and interorganellar signaling through lipid second messengers are critical for functional regulation. In the context of antigen presentation, a key lysosomal function in immune cells, the acidification of lysosomes by V-ATPase (stabilized by ATP6AP1 in HCC) is essential for proper antigen processing (85). Dysregulation of this acidification impairs antigen presentation and promotes an immunosuppressive environment, demonstrating how lysosomal pH, governed by ion pumps, directly communicates with and shapes the adaptive immune response (85). Therefore, future research must dissect the molecular machinery of lysosome- mitochondria contact sites in renal immune cells, examine how ER stress sensors like IRE1α modulate lysosomal cathepsin activity, and explore how metabolic signals from these organelles influence nuclear transcription factors (e.g., TFEB/TFE3) that govern lysosomal gene expression and epigenetic landscapes in kidney parenchymal cells during autoimmunity.

### 8.3 Biomarkers and clinical translation

The clinical translation of lysosomal checkpoint biology hinges on two parallel tracks: the discovery of non-invasive biomarkers to monitor disease activity and therapeutic response, and the rational design of clinical trials targeting specific lysosomal pathways, often in combination with existing therapies. Exploring lysosome-derived vesicles or specific enzymatic activities in the circulation represents a promising avenue for biomarker development. Lysosomal components released from injured or activated cells can serve as peripheral indicators of renal pathology. For example, the lysosomal protease Cathepsin C (CTSC) shows widespread dysregulation across cancers and correlates with immune infiltration and patient prognosis, supporting its potential as a circulating prognostic biomarker (83). Similarly, the lysosomal membrane protein LAMP-1 (CD107a) is a well- established marker of immune cell degranulation and its detection has been used to assess natural killer (NK) cell function in diseases like non-alcoholic fatty liver disease (NAFLD) (87). In NAFLD, the frequency of dysfunctional NK cell subsets characterized by altered expression of Siglec-7 (a sialic acid-binding receptor) correlates with disease, suggesting that immune cell surface phenotypes reflecting lysosomal/degranulation potential could be informative (87). In glomerulonephritis, targeted nanoparticle therapies designed to escape lysosomes and deliver payloads to mesangial cells have shown efficacy in reducing inflammatory cytokines like TNF- α and TGF-β1 (88). The success of such targeted approaches suggests that monitoring the levels of these cytokines, whose production may be influenced by lysosomal signaling, could serve as pharmacodynamic biomarkers for treatment response. The second critical direction involves designing clinical trials that target specific lysosomal checkpoints and evaluating their synergy with conventional immunosuppressants. The immunosuppressive tumor microenvironment in HCC, driven in part by ATP6AP1-mediated lysosomal acidification and pyroptosis, presents a rationale for combining ATP6AP1 inhibition with immune checkpoint inhibitors (ICIs) like anti-PD-1/PD-L1 antibodies to overcome resistance (85). This concept of combination therapy is bolstered by proof-of-concept studies showing that nanoparticle-delivered PD-1/PD-L1 inhibitors can enhance immune cell-mediated killing of cancer cells, partly by affecting lysosomal parameters (89). In autoimmune contexts, restoring defective lysosomal checkpoints in autoreactive T cells, akin to the anomalies seen in RA, could be a curative strategy if applied during asymptomatic autoimmunity (80). Furthermore, modulating the lysosome- dependent process of autophagy with agents like rapamycin, in combination with standard-of-care immunosuppressants, may be tested in progressive kidney fibrosis, given autophagy’s dual role in renal protection and pathology (86). For instance, enhancing protective autophagy in tubular cells while inhibiting maladaptive processes in fibroblasts could be a nuanced therapeutic goal. Clinical trials could initially focus on patients with active lupus nephritis or ANCA-associated vasculitis, measuring candidate lysosomal biomarkers (e.g., plasma cathepsin activity, LAMP-1+ extracellular vesicles, or specific autoantibodies against lysosomal proteins) alongside traditional renal function tests and histology to validate their utility and guide personalized, lysosome-targeted adjunctive therapy.

## 9 Conclusion

The lysosomal checkpoint represents a sophisticated and dynamic regulatory network that profoundly influences the initiation and progression of renal autoimmune diseases. This network operates across multiple levels, orchestrating processes ranging from the initial processing of autoantigens to the systemic metabolic dysregulation that characterizes these conditions. By integrating signals from antigen presentation, inflammatory pathways, cell death mechanisms, and metabolic states, lysosomes serve as central hubs that determine the direction of immune responses. Their dysfunction is not merely a bystander effect but a critical driver in breaking immune tolerance and fueling disease pathogenesis. This review has explored the multifaceted roles of lysosomes in renal autoimmunity, highlighting their involvement in key cellular events and their potential as therapeutic targets.

From an expert perspective, the development of the lysosomal checkpoint concept marks a significant advancement in our understanding of renal autoimmune diseases. It shifts the focus from isolated pathogenic mechanisms to an integrated view where lysosomes act as coordinators of immune and metabolic cross-talk. This paradigm emphasizes that disruptions in lysosomal function—whether in antigen- presenting cells, renal parenchymal cells, or systemic immune cells—can converge to promote autoimmunity. For instance, impaired lysosomal degradation may lead to the accumulation of autoantigens, while altered lysosomal signaling can exacerbate inflammatory responses or disrupt cellular homeostasis. The challenge lies in balancing the diverse research perspectives that have emerged. Some studies highlight the cell-specific roles of lysosomes, such as in podocytes or tubular epithelial cells, while others focus on their systemic immunomodulatory effects. Reconciling these views requires recognizing that lysosomal functions are context- dependent; their impact varies across different renal microenvironments and disease stages. Future research must therefore adopt a more nuanced approach, employing advanced techniques like single-cell sequencing and spatial transcriptomics to dissect the precise mechanisms of lysosomal checkpoints in specific cellular niches.

The therapeutic implications of targeting lysosomal pathways are equally promising and complex. Traditional agents like hydroxychloroquine have long been used in autoimmune diseases, partly due to their lysosomotropic effects, which modulate antigen processing and immune activation. These drugs validate the therapeutic value of lysosomal intervention but also reveal limitations, such as off-target effects and variable efficacy. Emerging strategies aim to overcome these drawbacks by developing more specific modulators of lysosomal enzymes, membrane proteins, or associated metabolic pathways like mTOR and autophagy. For example, inhibitors of specific lysosomal proteases or enhancers of lysosomal acidification could offer targeted control over autoantigen presentation. Similarly, modulating autophagy—a process intimately linked to lysosomal function—holds potential for restoring cellular homeostasis in renal cells. However, balancing the enthusiasm for these novel interventions with clinical practicality is crucial. While preclinical studies show promise, translating these findings into effective therapies requires careful consideration of pharmacokinetics, safety profiles, and patient stratification. Moreover, the dual role of lysosomes in both promoting and suppressing autoimmunity—depending on context—necessitates therapies that can precisely tune lysosomal activity rather than broadly inhibit or activate it.

Looking ahead, the field must prioritize several key directions to harness the full potential of lysosomal checkpoints in managing renal autoimmune diseases. First, there is a need to deepen our mechanistic understanding of how lysosomal networks interact with other cellular systems, such as the endoplasmic reticulum or mitochondria, in disease contexts. This will require interdisciplinary collaborations integrating immunology, cell biology, and metabolomics. Second, identifying reliable biomarkers of lysosomal dysfunction could revolutionize disease diagnosis and monitoring. For instance, lysosomal enzyme activities or specific metabolite profiles in urine or blood might serve as early indicators of disease onset or progression. Third, advancing targeted therapies will depend on innovative drug delivery systems that can selectively reach renal cells or immune compartments without systemic toxicity. Nanotechnology-based approaches or cell-specific targeting ligands could enhance the precision of lysosomal modulators. Finally, clinical trials must be designed to account for the heterogeneity of renal autoimmune diseases, potentially stratifying patients based on lysosomal pathway signatures to improve therapeutic outcomes.

*A summarized overview of these checkpoints, their disease relevance, and evidence levels is provided in* **Table 1**. In conclusion, the lysosomal checkpoint embodies a pivotal regulatory axis in renal autoimmunity, offering a unifying framework to explain diverse disease manifestations. Its study bridges gaps between immune dysregulation, metabolic alterations, and tissue damage, providing a holistic view of pathogenesis. By continuing to explore its intricacies—through balanced research that respects both cellular specificity and systemic integration—we can move toward more effective and personalized management strategies. The journey from bench to bedside will demand rigor and creativity, but the potential to transform the care of patients with renal autoimmune diseases makes this a compelling and vital pursuit in modern medicine.

**Table 1:** Summary of lysosomal checkpoints in renal autoimmunity.

Lysosomal checkpoint axis	Specific mechanism/molecule	Renal disease context	Primary cell type(s)	Evidence level
Proteolytic/Antigenic	Cathepsin S (CTSS) activity	Lupus Nephritis	Dendritic Cells, B cells	Preclinical (Murine Model)
Proteolytic/Antigenic	Cathepsin C (CTSC) activation	ANCA Vasculitis	Neutrophils	Genetic Association/Mechanistic
Signaling/Metabolic	mTORC1 Hyperactivation	Lupus Nephritis	T cells	Clinical & Preclinical Evidence
Signaling/Metabolic	Defective Autophagy Flux	Lupus Nephritis	Podocytes, T cells	Clinical & Preclinical Evidence
Structural/Integrity	Lysosomal Membrane Permeabilization	Crystal Nephropathy/Potential in LN	Tubular Epithelial Cells	Mechanistic Model/Extrapolated
